# Paleoamerican Diet, Migration and Morphology in Brazil: Archaeological Complexity of the Earliest Americans

**DOI:** 10.1371/journal.pone.0023962

**Published:** 2011-09-14

**Authors:** Sabine Eggers, Maria Parks, Gisela Grupe, Karl J. Reinhard

**Affiliations:** 1 Laboratório de Antropologia Biológica, Departamento de Genética e Biologia Evolutiva, Universidade de São Paulo, Instituto de Biociências da USP, São Paulo, SP, Brazil; 2 Anthropology Department, Texas A&M University, College Station, Texas, United States of America; 3 Ludwig-Maximilians-University Munich, Graduate School Life Science Munich, Planegg-Martinsried, Germany; 4 School of Natural Resources, University of Nebraska – Lincoln, Lincoln, Nebraska, United States of America; Paleontological Institute of Russian Academy of Science, United States of America

## Abstract

During the early Holocene two main paleoamerican cultures thrived in Brazil: the Tradição Nordeste in the semi-desertic Sertão and the Tradição Itaparica in the high plains of the Planalto Central. Here we report on paleodietary singals of a Paleoamerican found in a third Brazilian ecological setting – a riverine shellmound, or sambaqui, located in the Atlantic forest. Most sambaquis are found along the coast. The peoples associated with them subsisted on marine resources. We are reporting a different situation from the oldest recorded riverine sambaqui, called Capelinha. Capelinha is a relatively small sambaqui established along a river 60 km from the Atlantic Ocean coast. It contained the well-preserved remains of a Paleoamerican known as Luzio dated to 9,945±235 years ago; the oldest sambaqui dweller so far. Luzio's bones were remarkably well preserved and allowed for stable isotopic analysis of diet. Although artifacts found at this riverine site show connections with the Atlantic coast, we show that he represents a population that was dependent on inland resources as opposed to marine coastal resources. After comparing Luzio's paleodietary data with that of other extant and prehistoric groups, we discuss where his group could have come from, if terrestrial diet persisted in riverine sambaquis and how Luzio fits within the discussion of the replacement of paleamerican by amerindian morphology. This study adds to the evidence that shows a greater complexity in the prehistory of the colonization of and the adaptations to the New World.

## Introduction

**Figure 1:**
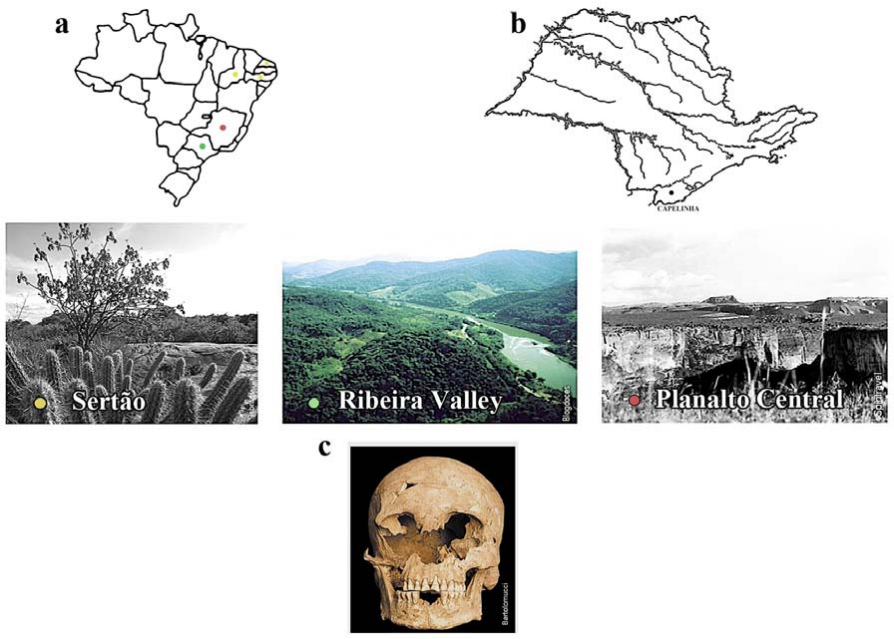
Location and typical landscapes where Paleoamericans were found in Brazil. (a) yellow- in the arid Sertão (including all or parts of the states Piauí, Pernambuco and Rio Grande do Norte); red- in the Planalto Central (state of Minas Gerais), and now, green – in the Ribeira valley (state of São Paulo). (b) main rivers of the state of São Paulo and the riverine location of the site Capelinha. (c) Burial II from Capelinha: Luzio.

A diversity of Paleoindian cultures thrived in Brazil during the transition to the early Holocene. The best known are the Tradição Nordeste (Northeastern Tradition) from the arid northeastern states of Piauí, Pernambuco, and Rio Grande do Norte. This semi-desertic region is called the Sertão (Figure 1a - yellow). The oldest skeletons from this region date to 11,120–11,025 years before present or yBP [1]. These groups subsisted partly on small animals hunted with bifacial projectile points, used rock shelters, developed clay artifacts and represented ritual and daily scenes in complex rock art panels [1]. Another early Holocene culture settled in the central high plains, the Planalto Central (Figure 1a - red). These sites are associated with a typical small and unifacial curated lithic industry [2], called the Tradição Itaparica, that expanded over about two million square km of the tropical Brazilian savannah from about 12,000 to 6,500 yBP [3], [4], [5], [6].

The skeletons from Tradição Itaparica and Tradição Nordeste date between 11,500 and 7,500 years ago and are characterized by a skull shape unlike later Native Americans [7]. This paleoamerican morphology is sometimes called “pre-mongoloid”, because the skulls are morphologically different and older than Native Americans of the middle and late Holocene. More specifically, these Paleoamericans show a more generalized “australo-melanesian” cranial morphology [7]. Using different methods and a distinct set of osteological collections for comparison, the Brazilian Paleoamericans, such as those form Lagoa Santa (who lived in the Planalto Central), are alternatively reported to exhibit strong morphological affinities with the prehistoric Jomon of Japan and the recent Fuegians [8].

Levy Figuti of the Museu de Arqueologia e Etnologia, Universidade de São Paulo and his team discovered a paleoamerican burial in a third Brazilian ecological setting [9], [10]. The burial was found in the riverine shellmound called Capelinha, in the southeastern Brazilian state of São Paulo (Figure 1b and c - green). Unlike other published paleoamerican skeletons, the collagen from the burial was well preserved and could be analyzed for stable carbon and nitrogen isotopes. It provides the oldest collagen-based evidence of paleodiet for a Brazilian Paleoamerican so far reported for an international readership.

More than a thousand shellmounds, or sambaquis, have been documented in Brazil. Most of them are coastal [11], [12]. They are found along almost the entire Brazilian shore where lagoonal areas with estuarine environments promoted a great diversity of fish, molluscs and crustaceans that favored sambaqui settlement [13]. About 300 radiocarbon dates attest that coastal sambaquis begun around 6,500 yBP [14], with their apogee between 5,000 and 3,000 yBP [11], [14]. However, there are three more ancient dates (ranging around 8,000 BP), indirectly confirmed through natural gamma radiation measurements of sand deposits [14]. The most famous coastal sites are monumental sambaquis, some of which are 400 meters long and up to 30 meters high [11]. Generally, sambaquis were used as dwelling and burial places [15], [16], [17], [18], [13].

Fluvial, or riverine sambaquis are located along inland rivers and are usually smaller than coastal sambaquis [9], [19]. The biggest concentration of fluvial sambaquis is in the state of São Paulo in the Ribeira de Iguape Valley (Figure 1a and b – green), and are the focus of recent excavations [9], [10]. Although these riverine sambaquis are located about 60 km from the coast, marine artifacts have been found in some of them, attesting to contact between the riverine and coastal groups [9], [18]. The fact that they contain mollusc layers meant for some researchers that they were used coastal peoples moving inland, possibly because of population pressure near the seashore [19], [20], [21]. In addition to some marine remains typical of sambaquis, features of other cultures were also found in the Capelinha site, suggesting different occupations [9]. Therefore, Capelinha is also considered a multicomponential site [22].

With regard to early Holocene archaeology, riverine sambaquis are significant because some of them possess great antiquity [9], [10]. Rising sea levels of the early Holocene inundated the oldest coastal sambaquis [23], [24], but fluvial sambaquis did not suffer this fate. Therefore, they offer the possibility of discovering early Holocene adaptations.

## Materials and Methods

Among the Paleoamerican remains discovered in the sambaqui Capelinha, a complete adult male was exhumed and dated to 10,180 – 9,710 yBP (Figure 1c and Table 1 - [9], [10]. This great antiquity was totally unexpected, since it resembled the dates published by [19] that were discredited by many Brazilian archaeologist in the past. However, shell, charcoal and other bones from Capelinha were dated more recently [9], finally confirming this old age (Table 1).

**Table 1:** Dates of Luzio and other remains from the Capelinha site (modified from [9]).

Site Feature	Sample number	Sample Type	Depth (cm)	Conventional Date, BP	Calibrated Date, BP
Luzio	Beta 153988	Human Bone	0–30	8860±60	10180 − 9710
Midden	A 11239	Charcoal	90–100	8795±105	9879±198*
Midden	A 11236	Shells	80–90	8500±70	9497±37*
Burial V	Beta 184619	Human Bone	10–20	6090±40	7020 − 6850

*Calibrations from midden remains were calculated from http://www.calpal-online.de/.

Capelinha is one of about 30 riverine sambaquis discovered in the Ribeira de Iguape Valley and, like all fluvial sambaquis, it is composed of gastropod shells of terrestrial species, mainly of the genus *Megalobulimus* [9]. Capelinha is located in the southern part of the São Paulo state at an altitude of 310–320 m, as seen in Figure 1b (UTM 22J 0778967 / 7249040 –[24]).

Previously, an ancient Paleoamerican female skeleton was found in the Planalto Central region of Lagoa Santa. She was named Luzia, the first American [7]. Because our male skeleton showed morphological similarities with Luzia, he was called Luzio. Indeed, Luzio has morphologic affinities with Luzia based on craniometric analysis [10].

Luzio was in a primary burial as an articulated skeleton in flexed position found at 20 cm depth in a dark orange clay matrix on top of a thin shell layer [9]. He is a well- preserved, almost complete, and extremely gracile adult [10]. In life he was not more than 160 cm high, but his skeleton has marked muscle insertions and some osteoarthritic changes, indicating strong physical activity, according to widely applied methods [25], [26], [27]. His well preserved teeth are not as heavily worn as is typical for later coastal sambaqui people [28] and show a hunter-gatherer pattern of wear and four minute caries. The higher degree of tooth wear found in the frontal teeth as compared to the premolars and molars, suggest an abrasive diet, such as that often seen among hunter gatherers [29]. Unfortunately, Luzio's dental calculus is so faint that it precludes the mechanical detachment necessary for the recovery of plant microfossils that could provide insights into the family or species of plants consumed [30]. Furthermore, there is a probable agenesis of all third molars and paramasticatory wear of upper incisors (Figure 1c), indicating he used his teeth as tools. What exactly the activities were that he performed using his teeth are still unknown. Luzio further shows a rare and not yet fully examined craniosynostosis which did not affect the shape or size of his head [10]. Since there is no sign of chronic infectious disease or trauma, his cause of death could not be determined.

Some traces of sambaqui ritual can be seen in Luzio's grave. Like most later sambaqui dwellers or sambaquieiros, Luzio was buried in a flexed position, and was placed in an artificially prepared grave on top of a layer of shells located on a prominent land mark [9]. He was buried with offerings of polished bone artifacts, as well as a stone projectile point. Nevertheless, his grave also shows remarkable differences in comparison to later sambaqui dwellers because it was covered with clay instead of shell, and because it was placed on a natural landmark, a terrace above a stream, not in an artificial mound [9].

Luzio is the oldest excavated sambaqui dweller, or sambaquieiro, the oldest human so far discovered in the state of São Paulo, and one of the oldest skeletons from the Americas in general.

Carbon isotope ratios reflect the degree to which individuals, directly or indirectly, consume different types of plants, specifically C_3_, C_4_, and CAM plants [31], [32]. Calvin-Benson plants, or C_3_ plants, employ a 3-carbon molecule to break down carbon dioxide during photosynthesis, and discriminate against the slower-moving ^13^C isotope to a greater degree than the lighter ^12^C isotope. C_3_ plants include tubers, legumes, many shrubs and trees and show an average δ^13^C value of −26‰. Plants that use the Hatch-Slack photosynthetic pathway, or C_4_ plants, include tropical grasses such as corn. These plants use a 4-carbon molecule to break down carbon dioxide from the atmosphere. C_4_ plants have an average isotopic value of roughly −12.5‰ because they incorporate proportionately more of the heavier ^13^C isotope. Crassulacean Acid Metabolism plants, or CAM plants, have an isotopic value that lies in an intermediate range between C_3_ plants and C_4_ plants. These plants tend to exist in xeric environments, and include succulents and cacti [33], [34], [35].

Dietary inferences based on carbon isotope ratios must take into consideration the source of the biological tissue analyzed. δ^13^C values derived from bone apatite reflect the whole diet, as carbohydrates, proteins, and lipids are incorporated from the circulatory system as dissolved bicarbonate. δ^13^C values derived from bone collagen mainly reflect the protein portion of the diet [36], [37].

When used in conjunction with stable carbon isotopes, nitrogen isotopes allow the researcher to determine the trophic level of dietary protein. δ^15^ N values become enriched by 3‰ at each trophic level of the protein source such that the nitrogen isotope values of herbivores (such as deer), and carnivores (such as predatory fish), would differ significantly [38]. Due to the wide range of carbon sources that contribute to marine plants, marine organisms can have δ^13^C values that fall between C_3_ and C_4_ terrestrial plants. Using nitrogen and carbon isotopes together, marine sources of dietary protein, such as fish, can be distinguished from terrestrial sources of protein [32], [39], [40]. These dietary distinctions are crucial for studies involving prehistoric groups who may have exploited both marine and terrestrial resources.

Four well preserved proximal foot phalanges from Luzio were analyzed by two different laboratories, Geochron Laboratories and the Texas A&M University Stable Isotope Laboratory in the Department of Anthropology. At Geochron, the standard methods were employed and the results were used as control as well as for comparison (Table 2).

**Table 2:** Relevant isotopic data from Luzio.

	C,N Ratio	Collagen Yield	delta 15N Collagen	delta 13C Collagen	delta 13C Bioapatite	delta 13C Spacing Value
Geochron	-	-	12.1	−19.3	−13.2	6.1
Texas A&M	3.376	2.7	10.94	−20.23	-	-

At Texas A&M, a sample of a foot phalange weighing approximately 1 gram was mechanically cleaned with a file and placed in a sonicator, where it was cleaned in baths of distilled water, 95% ethanol, 100% ethanol, and acetone. Using a 0.25 M solution of hydrochloric acid (HCl), the bone sample was demineralized until the soft, organic fraction of the bone remained. The collagen pseudomorph was centrifuged thoroughly in distilled water until a neutral pH was reached. To remove humic contaminants, the pseudomorph was soaked in a 0.125 M solution of sodium hydroxide (NaOH) for 24 hours, and then centrifuged thoroughly in distilled water until pH neutrality was attained. The collagen pseudomorph was then solubilized in pH 3 water for three days in a 90°C oven. The dried collagen sample was extracted, weighed, and sent to Dr. Tom Boutton in the Department of Rangeland Ecology at Texas A&M University, where mass spectrometry was performed with a Carlo Erba EA-1108 interfaced with a ThermoFinnigan Delta Plus isotope ratio mass spectrometer operating in continuous flow mode.

Preservation of the sample was assessed using measurements of the carbon to nitrogen ratio (C,N ratio) and the percent yield of collagen. The sample provided a C,N ratio of 3.4, which falls within the acceptable range of 2.9 to 3.6, and a collagen yield of 2.7% which also falls within the parameters for acceptable collagen preservation [41], [42]. Isotopic analysis of the bone collagen revealed a δ^13^C value of −20.23‰ and a δ^15^N value of 10.94‰ (Table 2).

## Results and Discussion

Stable carbon isotope ratios taken from bone collagen (δ^13^C −20.23‰) and apatite (δ^13^C −13.2‰) confirm that Luzio subsisted on C_3_ plant and animal resources, and that neither marine nor freshwater protein contributed to a significant part of his diet. Previous study [43] reports that paleodietary interpretations are most robust when carbon isotope ratios from collagen and apatite are plotted against one another, rather than used independently or as a measurement of Δδ^13^C_CO-AP_. This method proves especially critical for environments that contain a mixture of C_3_, C_4_, and marine resources, or when an isotopic measurement seems to contradict the presumed dietary regime of an archaeological population [43]. However, little comparative data of this kind is available for this approach, so we rather preferred comparing Luzio's collagen results with those of other organisms and human groups, as seen below. The stable isotope results suggest that he consumed mainly C_3_ plant foods, as well as animals that relied on C_3_ terrestrial resources.

From the plants that existed in the Ribeira de Iguape Valley at the beginning of the Holocene [44], some might have been used by Luzio and his group. Ethnobotanical research shows the following Holocene plants and their utility in recent populations living in the region in which Luzio was found: *Maclura trinctoria* (fruit and dye), *Chrysophyllum inornatum* (fruit, oil, wood, and medicine), *Panicum pilosum* (seeds for food), *Senna multijuga* (seeds for beverage), and *Verbesina glabrata* as well as *Lonchocarpus muehlbergianus* (both used as medicine) [45]. It would be very interesting to test if microfossils from these plants are in fact present in the faint dental calculus that still persist on Luzio's teeth. However, this could only be carried out using the somewhat destructive but still very informative dental wash method [46], [47].

Although Luzio's skeletal remains were associated with a riverine sambaqui, there is little evidence that freshwater resources, such as fish or shellfish, contributed to a significant part of his diet. However, it is difficult to assess the probable dietary influence of shellfish consumption, since modern data on shellfish are heavily biased by sewage effluents which are absorbed by these animals as a nutrient source [48], [49], [50]. In general, fluvial nitrogen signals are distinct from marine such signals, whereby δ 15N-values up to 10‰ are encountered in estuarine and marine shell fish. Since phytoplankton belongs to the predominant food source for shellfish such as oysters [51], riverine shellfish preserve the terrestrial δ 15N signal [52]. Since also Luzio's collagen δ 13C is indicative of a largely terrestrial diet, the consumption of shellfish might well have contributed to his diet, but most probably not to a large extent which should have shown up in the δ 15N values. Freshwater fish will be characterized by rather high δ 15N values in its edible parts according to the species' dietary behavior, but will at the same time reveal depleted δ 13C signatures [53]. The collagen stable isotope values in the skeleton of Luzio are not indicative for a considerable proportion of fish or shellfish in the overall diet.

Instead, Luzio probably hunted local terrestrial animal resources, such as peccary, deer, and rodents, since the collagen δ^13^C and δ^15^N values for these faunal resources [54] fall within the isotopic range observed in Luzio's diet. In an isotopic analysis of archaelogical faunal remains taken from coastal sambaquis of Santa Catarina, southeastern Brazil, De Masi [54] reported collagen δ^13^C values of − 21.52‰ for *Tayassu sp.* (peccary), −18.72‰ for *Mazama americana* (red brocket deer), and −14.98‰ for *Hidroachaeris hidroachaeris* (capivara). δ^15^N ratios for *Tayassu* sp.and *Mazama americana* were reported as 3.64‰ and 4.73‰, respectively [54].

The carbon isotope values above suggest that red brocket deer and peccary were part of Luzio's diet. Indeed, zooarchaeological investigations in this site attest that the great majority of hunted animal protein consumed derived from medium sized terrestrial animals such as peccary, red brocket deer and small rodents such as the Brazilian guinea pig (*Cavia aperea*) [9]. This suggests two different hunting strategies, a systematic hunt of rodents and a more specialized search for ungulates at longer distances from the settlement [9]. The dietary use of small animals is consistent with other Paleoamericans from Brazil, such as those from Lagoa Santa [55], as well as with North American hunter-gatherer groups [56].

### Luzio in comparison to other human groups

**Figure 2:**
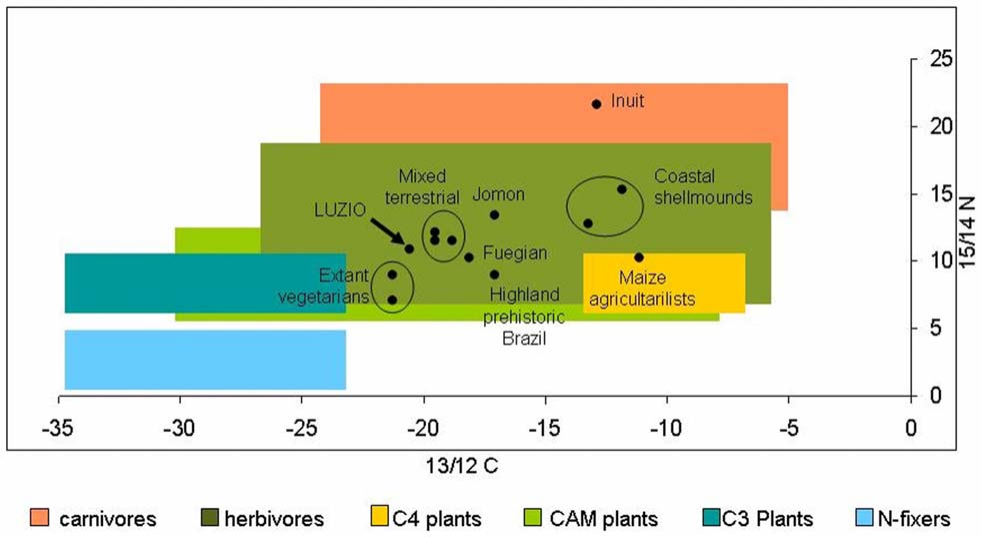
Isotopic signatures of Luzio (arrow - this study) and mean collagen isotopic singature values from selected prehistoric and extant human populations (13/12 C, and 15/14N) plotted on top of the isotopic variation of different organism groups. Modified from 58; 59; 60; 54; 61; 62; 63; 64; 65; 66; 67; 68– see Table 3.

The isotopic data shown herein confirm a mainly terrestrial diet for Luzio. When plotted against a background of values attributed to different organisms, Luzio's values fall within the intersection of herbivores and CAM plants (Figure 2) [57], [58], [59], [60], [61], [61], [63], [64], [65], [66], [67,68]. At first sight it seems curious that CAM plants (such as cacti, succulents, some bromeliae and orchids) were available for consumption in the Atlantic forest. However, the Atlantic forest complex shows one of the world's greatest biodiversity, including plants with a plurality of ecophysiological mechanisms enhancing fitness and survival [57], such as those described for CAM plants.

Luzio's paleodietary signatures are similar to those recently found for this and other humans exhumed from riverine shellmounds in the Vale do Ribeira region: the collagen isotopic values from individuals from the Capelinha, Moraes and Estreito sites (who lived between about 10,000 and 4,100 yBP) range from −19.2 to −21.4 for δ^13^C and from 9.6 to 11.9 for δ^15^N [69], also suggesting mainly terrestrial diet. Typical terrestrial dietary values were also found for the Paleoamericans from the Planalto Central (Lapa do Santo, Lapa das Boleiras and Santana do Riacho, aged between 10,000 to 6,000 y BP) with mean δ^13^C and δ^15^N collagen values of −19.58±1.6 and 8.19±1.7, respectively [70].

In comparison to some other selected human groups, Luzio scores between extant ovo-lacto vegetarians from Denmark and England and prehistoric groups with mixed terrestrial diet, such as the forest people from Santarém (Brazil), the people from Çatalhoyuk with their plant cultivation, sheep and goats, as well as the ancient Nubians whose diet was based on C4 plants and cattle (Table 3 and Figure 2). This shows that there are important limits and overlaps between different life-styles, even if all these different groups can be classified as sharing a “mixed terrestrial” diet.

**Table 3:** Mean collagen δ13C and δ15N of selected extant and prehistoric human groups.

site/group	age	main subsistence	Mean δ13C	SD	Min	Max	Mean δ15N	SD	Min	Max	Reference	Country	Classification
Danish vegetarians	extant	no animal protein	−21,1	0,3	−21,7	−20,9	9,7	0,3	9,4	10,4	[59]	Denmark	Extant Vegetarians
Ovo-lactoVegetarians Oxford	extant	ovo-lacto-vegetarians	−21	0,3	NA	NA	6,9	0,5	NA	NA	[64]	England	Extant Vegetarians
Luzio	10180−9710 BP	terrestrial	−20,23	NA	NA	NA	10,94	NA	NA	NA	this study	Brazil	Luzio
Santarém Tropical forest	extant	local mixed terrestrial	−19,1	1	NA	NA	11,7	0,3	NA	NA	[63]	Brazil	Mixed terrestrial diet
Çatalhoyuk, adults, south	8000−7000BP	plant cultivation, goat, sheep	−19,09	NA	NA	NA	11,17	NA	NA	NA	[66]	Turkey	Mixed terrestrial diet
Selk'nam Fuegians	1500−500BP	terrestrial (guanaco?), C4 plants	−18,6	NA	NA	NA	9,9	NA	NA	NA	[68]	Tierra del Fuego	Mixed terrestrial diet
Ancient Nubians, Kerma	4450−4000BP	terrestrial, C4 plants, cattle	−18,2	NA	NA	NA	11,1	NA	NA	NA	[61]	Sudan	Mixed terrestrial diet
Terras Altas Santa Catarina	1610−1182BP	terrestrial	−16,96	2,94	−10,77	−20,64	8,65	0,95	10,37	7,64	[54]	Brazil	Highlanders Brazil
Jomon from Ota	5000−4000 BP	marine fish+terrestrial	−16,7	1,1	NA	NA	12,9	1,8	NA	NA	[62]	Japan	mixed terrestrial/marine
Matjes River shellmound	7500−2000 BP	marine & terrestrial	−13,6	1,5	−17,6	−9,1	13,2	1,8	6,8	17,7	[67]	South Africa	Coastal shellmound
Dorset Sadlermiut	800BC−1000AD	seals, walrus, whale	−13,3	0,3	NA	NA	20,9	0,5	NA	NA	[60]	Canada	Inuit
Coastal Sambaquis Santa Catarina	4070−1590BP	marine, fish, shellfish	−11,87	1,05	−9,28	−13,35	15,76	1,72	18,47	12,39	[54]	Brazil	Coastal shellmound
Bennett/Woodbridge	1275−1500AD	maize agriculture	−11,58	NA	NA	NA	11,13	NA	NA	NA	[58]	Canada	Maize agriculturalists

For a sambaqui dweller, Luzio's dietary signatures are strikingly different from the diet consumed by classic coastal shellmound people, who subsisted mainly on marine resources - Figure 2 and Table 3) [54], [71], [13], [67]. This means that he was very well adapted to the Atlantic forest environment in which he lived and died.

However, as a Brazilian Paleoamerican [10], Luzio also shows an expected uniqueness. Luzio's morphology can be claimed to be similar to either Australo-Melanesians [7], or to peoples such as the Jomon from Japan or even the Fuegians from Tierra del Fuego [8]. The isotopic signatures of these peoples, are obviously different, since they subsisted on totally different strategies and also enjoyed a distinct way of life (Figure 2 and Table 3).

Thus, Luzio, a sambaqui representative, stands out through his antiquity, his paleoamerican morphology and now with his terrestrial diet.

### Terrestrial diet and coastal contacts

Luzio's presence shows that paleohuman adaptation in the Capelinha site 10,000 years ago was based on terrestrial resources found along river habitats, and not mainly on fish as found in coastal sambaquis [54], [13], [18], [71]. The presence of shells, shark teeth and artifacts from the coast in the riverine shellmounds in general, as also in the Capelinha site [9], indicates that there was contact of these inland peoples with groups living at the coast; an observation made earlier by Neves [21]. Thus, riverine sambaquis could represent vestiges of migrations [19], seasonal trips [20], or even trade from the coast to the inland and back again. The route that could have made these trips or migrations through the otherwise steep and hostile Serra do Mar less strenuous is that following the Ribeira valley [72], [73]. Therefore, one could imagine that Luzio was part of a very large ocean-river sambaqui population that collected and traded a variety of necessities along waterways. The reliable supply of resources, especially at the coast, would have allowed sambaqui settlements to expand and stabilize very early in time. This predictability of resources resulted in the continuation of sambaqui life for probably more than 8,000 years along almost the entire Brazilian coast and, as we see also in the case studied herein, along some of the most important river valleys [11], [13].

Thus, Luzio with his paleoamerican morphology, terrestrial diet and cultural exchanges with coastal people seems to represent a fundamental key element for the better understanding of the microevolutionary processes that occurred in the transition to the early Holocene in southeastern Brazil.

### Where did Luzio's community come from?

Luzio is the first discovery of an early Holocene Paleoamerican outside the drier regions of central and northeastern Brazil and he is the southernmost Paleoamerican found in Brazil. This adds further support to the idea of the variability of Paleoamerican adaptation. Maritime technologies associated with paleoamerican dates of 12,100 to 11,200 yBP were also found in California's Channel Islands [74]. Luzio's associations with maritime artifacts and shell layers supports the hypothesis that paleohumans arrived in Brazil through coastal and riverine migration following both the Pacific as well as the Atlantic coast, to later colonize the Planalto Central [19], [75].

Curiously, however, the oldest evidence that suggests the use of boats in Brazil comes from the interior: a rock painting dated to between 12,000 and 6,000 yBP [76]. Although discredited in the past, the hypothesis of coastal migration from Asia to the New World [8], harvesting kelp forests on the Pacific shore [77], leaving evidences of seafaring and fishing technologies as soon as 12,000 years ago in Ice Age Santa Rosa island, California [74] and seaweed from distant beaches used for food and medicine in Monte Verde, Chile by 14,220−13,980 yBP [78] is being increasingly supported. Early Holocene riverine sambaquis in Southeastern Brazil would then represent coastal populations moving inland.

Alternatively, hunter gatherers coming from the inland, such as perhaps the slightly older Lagoa Santa people [7], could have migrated from the Planalto to the coast leaving riverine sambaquis along waterways as their testimony to their migration [79].

These two apparently contradictory hypotheses (migrations from the coast inland versus from the Planalto to the coast) can, however, be combined. Following Callipo [80], the Ribeira valley witnessed migration waves from and to the coast due to the rise and fall of the relative sea level occurred during the last 12,000 years [81], [23]. According to this model [80], interior hunter gatherers migrated towards the coast via the Ribeira valley when the sea level, 12,000 years ago, was much lower than today. With the rise of the sea level occurring between 12,000 and 9,000 yBP, these hunter-gatherers learned to exploit marine resources and the fishermen known as coastal sambaqui people developed. At the same time some of these maritime groups moved up the Ribeira valley originating the riverine sambaquis. Between 9,000 and 5,000 years ago the sea level rose above today's level, submerging some of the lower and older coastal shellmounds.

In case sambaquis older than 10,000 yBP will be discovered at the Brazilian seashore, a coastal origin of Luzio could be confirmed. So far, however, the origin of his ancestors is still mysterious.

In this context, the Capelinha site has another special significance. It shows a more complex stratigraphy in comparison to other riverine sambaquis (including not only shell layers, but also sand and charcoal lenses, as well as numerous projectile points from the Umbu tradition), higher investments in the care for the death and was constructed and occupied during a longer time span [9], [22]. This means the Capelinha site is multifunctional showing an overlap of distinct occupations [9], [22]. People living in the Ribeira valley would have competed for settlement sites, such as this one, and occupied them repeatedly in the search of the few spots in the Serra do Mar that are adequate for settlement purposes [22].

### Continuity of terrestrial diet in riverine sambaquis

Indeed these multiple occupations of the Capelinha site show that this spot of the Atlantic forest was the settlement and home of people during almost 8,000 years [9]. Other regions in the vicinity were also occupied, but the apogee of riverine sambaquis at the Ribeira valley seems to have occurred only about four thousand years after Luzio died [9], [69]. One of these sites is the already mentioned Moraes riverine sambaqui. Moraes, also excavated by the team headed by Levy Figuti, yielded the best osteologic collection of riverine shellmounds from Brazil. Much more recent (4,500 to 5,900 yBP - [9]) than Luzio from Capelinha, the people from Moraes show some similarities and some striking differences in comparison to the coastal sambaqui people. Bioanthropological similarities between Moraes and coastal sambaquis include [82]: a) equally high prevalence of auditory exostosis indicating frequent aquatic activities; b) significantly more frequent osteoarthroses in upper than lower limbs suggesting low walking distances; c) cranial and dental morphological affinities suggesting intense gene flow between coastal groups and the people from Moraes [73], [83]; and d) low frequencies of violent trauma, as if competition for resources, territory or perhaps mates was rare. However, there are also important differences: Moraes subsisted on a much broader protein diet and consumed more cariogenic food, but showed a stature even smaller than coastal groups, which perhaps could be the effect of frequent gastrointestinal diseases during childhood [82]. Thus, despite the contact between this riverine shellmound and coastal sambaquis suggested by gene flow, marine artifacts [9], as well as treponematoses in both sites [84], the people at Moraes were well adapted to local conditions, as shown by their terrestrial diet [69].

Until other skeletons are exhumed from Capelinha or other riverine sambaquis of the same antiquity in the Ribeira region, some of the inferences made on the way of life of the later people from Moraes may also apply to the life-style of Luzio's community. However, Luzio was a Paleoamerican and the people from Moraes were typical Amerindians [73]. So what was the fate of the paleoamerican morphology?

### Replacement of Paleoamerican by Amerindian morphology

In his long search for the peopling of the New World using craniometry, Neves and his colleagues [75], [10], [85] suggest that the paleoamerican morphology was replaced by the amerindian or mongoloid morphology between 8,000 and 3,000 yBP. On the other hand, because Paleoamericans show the same mtDNA haplogroups as Amerindians [86] and since farmers are usually brachycephalic, while hunter- gatherers are dolicocephalic [87], changes in diet and food preparation [88], as well as ecological specificities could explain the morphological differences between Paleoamericans and Amerindians [86].

In this background, Luzio fits well as a Paleamerican with his hunter-gatherer dental wear and terrestrial diet. But the later Amerindian sambaqui people, following this interpretation, should all have to have had a less abrasive, more processed and smoother diet, not only composed of marine resources, but perhaps also based on domesticated plants. Until now, however, bioarchaeological evidences of higher caries prevalence that could indicate plant domestication exist for only very few coastal sambaquis [89], [82].

Luzio stands out as representative of the first Paleoamerican people in what is known today as Brazil with strong ties to the coast. This had not been the case with the other evidences of paleoamerican morphology reported until now.

This, as well as other recent evidences, such as the similarity of the Jomon with the Paleoamericans in central Brazil [8], the transhumance between coastal and interior areas [78], the cultural and possibly also genetic exchanges of Paleoamericans and shellmound dwellers [73] as well as the growing reports on coastal migration [74], [77], [78] show a greater complexity of the history of migration to and the colonization of the New World. The scarcity of osteological collections dated to between 10,000 to 5,000 yBP that could confirm the existence of mixed paleoamerican and amerindian morphology represent a fruitful endeavor for new excavation campaigns in South America.

### Outlook

Luzio, with his paleoamerican morphology and antiquity [10], [9], his terrestrial diet (this study and Plens [69]), and some cultural features resembling coastal sambaquis [9] attests to intense contacts between people from the inland and the coast living in today's southeastern Brazil. Maritime artifacts, such as shark teeth buried with Luzio [9] suggest trade with an as yet undocumented coastal population. Today, the archaeological site where Luzio was unearthed lies 60 km from the coast. However, 10,000 years ago the sea level was lower [23] and so the minimal distance to the Atlantic must have been longer, augmenting the significance of the cultural ties with the coast. Then the water level rose several meters in mid-Holocene times and possibly inundated the oldest coastal sambaquis [23], [80]. Nevertheless, the discovery of Luzio makes us optimistic that further excavations of riverine shellmounds will reveal other evidence of the earliest Americans.
